# Interfacial Engineering-Free Microfluidics: Toward a Mild and Cost-Effective Strategy for Surfactant- and Demulsifier-Free Hydrogel Microsphere Fabrication

**DOI:** 10.3390/mi16070733

**Published:** 2025-06-22

**Authors:** Qing Qin, Yu Zhang, Yubei Wei, Jinnuo Lv, Meiling Tian, Yuanyuan Sun, Wei Fang, Xingjian Huang, Jianglin Li, Yifeng Su, Xiaoliang Xiang, Xing Hu, Zhizhi Zhou

**Affiliations:** 1 College of Biological and Food Engineering, Huaihua University, Huaihua 418008, China; 17803104182@163.com (Q.Q.);; 2 Hunan Provincial Higher Education Key Laboratory of Intensive Processing Research on Mountain Ecologcal Food, Huaihua 418000, China; 3 Key Laboratory of Research and Utilization of Ethnomedicinal Plant Resources of Hunan Province, Huaihua 418000, China

**Keywords:** hydrogel microsphere, microfluidics, demulsifier-free, surfactant-free, droplet formation, non-lithography

## Abstract

This study proposes a simple yet versatile microfluidic strategy for fabricating monodisperse alginate hydrogel microspheres using a symmetric flow-focusing device. The system integrates three key innovations: (1) Cost-effective mold fabrication: A paper-based positive master replaces conventional SU-8 photoresist, significantly simplifying device prototyping. (2) Surfactant-free droplet generation: Alginate hydrogel droplets are formed at the first flow-focusing junction without requiring interfacial stabilizers. (3) In situ solidification with coalescence suppression: Acetic acid-infused corn oil is introduced at the adjacent junction, simultaneously triggering ionic crosslinking of alginate via pH reduction while preventing droplet aggregation. Notably, the hydrogel microspheres can be efficiently harvested through oscillatory aqueous phase separation, removing post-fabrication washing steps (typically 6–8 cycles for surfactant and oil removal). This integrated approach demonstrates exceptional advantages in fabrication simplicity, process scalability, and operational robustness for high-throughput hydrogel microsphere production.

## 1. Introduction

Alginate hydrogel microbeads have found extensive applications, spanning from academic research to industrial production [1,2]. Traditional mass-production methods of alginate microcapsules commonly rely on mechanical stirring or external gelation-based mixing [3,4,5]. Nevertheless, these techniques frequently yield microcapsules with diverse morphologies and sizes, hampering their seamless application across various fields. In contrast, microfluidic techniques present an alternative route for generating monodisperse droplets with precisely tailored morphologies and dimensions [6,7,8,9], thus surmounting the technological hurdles associated with the above-mentioned applications. Chaotic mixing of alginate solution and calcium ions at T-junctions in microfluidic devices frequently induces channel clogging due to premature microgel cross-linking within confined channels [7]. To overcome this, calcium carbonate (CaCO_3_) nanoparticles have been used to produce uniformly sized microgels. Under acidic conditions, water-insoluble Ca^2+^ can be released into the alginate solution post–emulsification [6]. However, the heterogeneous distribution of large CaCO_3_ particles poses challenges, resulting in non-uniform cross-linking within the formed microbeads. To address this, Weitz’s group demonstrated the use of a water-soluble calcium–ethylenediaminetetraacetic acid (calcium–EDTA) complex to regulate calcium ion delivery. This approach enables excellent homogeneity in microgel formation without causing non-uniform gelation [8,9,10]. However, the fabrication of cross-linked microgels is a multi-stage process, demanding significant labor. To achieve labor-saving microbead production, Lin’s group developed an integrated microfluidic system comprising two functionally distinct chips. This platform utilizes a calcium-EDTA complex as a precursor to generate high-uniformity hydrogel microbeads, while enabling in situ formation and automated cross-linking for three-dimensional cell culture applications [11]. The Moulton group demonstrated a substantial enhancement in alginate-based biomolecule encapsulation efficiency by optimizing flow dynamics and polymer concentration ratios on a conventional microfluidic platform. Their approach utilized a surfactant-demulsifier co-stabilization system (Picosurf™ (Selins Biotechnology Co., Ltd., Jinan, China)/C_8_F_15_CH_2_OH) to achieve interfacial stability under physiologically relevant conditions, thereby enabling precise microbead formation while preserving biocompatibility [12]. However, similar studies in this field predominantly rely on costly chemical agents, including perfluorinated polyether-polyethylene glycol (PFPE-PEG) [11,12,13,14], Picosurf™ [12], neat 008-fluorosurfactants [13], Krytox–PEG–Krytox triblock copolymers [15], span 80 [2,16,17,18,19,20], polyglycerol polyricinoleate (PGPR) surfactants [21] for monodisperse emulsion generation, and 1H,1H,2H,2H-perfluoro-1-octanol (PFO) [2,11,12,13,14,15,16] for demulsification. Notably, few methodologies have addressed the critical need for cost-effective, low-reagent-consumption platforms compatible with resource-limited settings. Particularly, cell encapsulation, drug delivery, and tissue engineering, emphasizing how current demulsification-dependent production methods limit biomedical applicability due to solvent toxicity and low efficiency. This directly motivates our solvent-free microfluidic approach.

In this study, we present a novel approach for fabricating homogeneous alginate microbeads using a symmetric flow-focusing configuration integrated within a PDMS (polydimethylsiloxane) microfluidic device (Midland, MI, USA). This innovative methodology addresses several critical challenges: (i) A paper-based positive master replaces conventional SU-8 photoresist, significantly simplifying device prototyping. (ii) The implementation of pure oil as the continuous phase enables economical generation of monodisperse alginate hydrogel droplets, effectively eliminating the necessity for surfactant additives. (iii) The incorporation of acetic acid significantly mitigates droplet coalescence in the oil medium, thereby facilitating scalable production of uniform microbeads. (iv) The developed process allows for efficient separation of microspheres from corn oil through an oscillatory aqueous phase, completely removing the dependency on demulsifier phases.

## 2. Materials and Methods

### 2.1. Materials and Apparatus

All chemicals and materials were obtained from commercial sources: corn oil (Yihai Kerry Arawana, Yueyang, China), EDTA disodium salt (Beyotime Biotechnology, Shanghai, China), PDMS and initiators (Dow Corning, Midland, Mi, USA), calcium chloride (Xilong Scientific, Shantou, China), acetic acid (Chron Chemicals, Chengdu, China), and the self-adhesive paper (Kailiwen Business Co., Ltd., Chengdu, China). Fluid delivery was controlled using two programmable syringe pumps (Model XFP01-BD, Suzhou Xunfei Scientific Instruments Co., Ltd., Suzhou, China), equipped with 5 mL syringes (Model 0.6X26TWLB, Jiangxi Fenglin Medical Equipment Co., Ltd., Fuzhou, China). Solutions were infused through PTFE tubing (inner diameter [ID]: 0.35 mm, outer diameter [OD]: 0.5 mm; Woer Co., Shenzhen, China) at constant flow rates. The sodium alginate solution was infused at 2 μL/min, while the corn oil/acetic acid mixture phase was tested at discrete flow rates of 30, 35, 45, and 50 μL/min to establish flow-dependent behavior. These parameters ensure reproducible flow dynamics in our microfluidic platform. The material properties of the working fluids have been provided in Table 1, including density, apparent viscosity and interfacial tension. Kinematic viscosities (ν) were determined at 25 °C using a Rapid ViscoAnalyser (2 Plus, Thermo fisher scientific, Waltham, MA, USA). Dynamic viscosity (η) can be calculated by η=ρ×ν. The interfacial tension (γ) of the different fluids was tested by Surface tension meter (BZY-1, Hengping instrument, Shanghai, China).

### 2.2. The Fabrication of the PDMS Microfluidic Device

**Figure 1:**
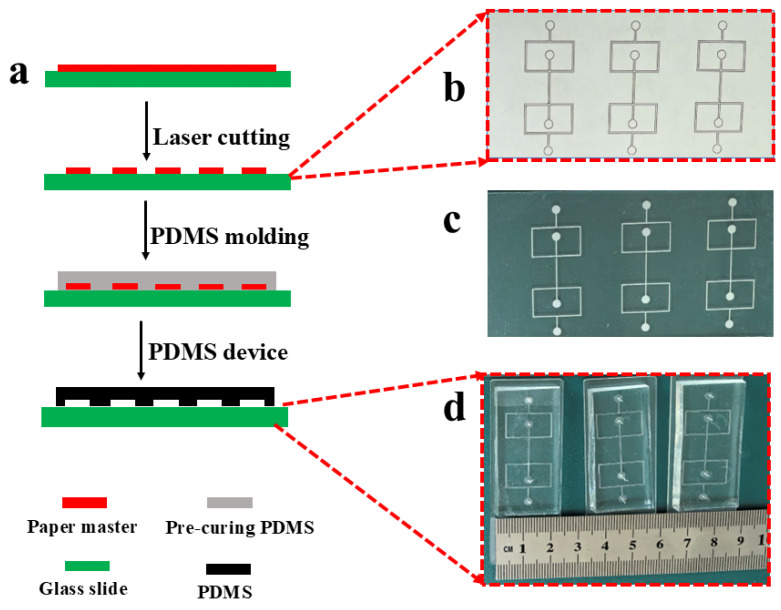
Fabrication procedure of the PDMS-based microfluidic device. (**a**) Schematic of the microfluidic device’s flow-focusing pattern, designed using a simple, low-cost method. (**b**) Multiple symmetric flow-focusing patterns laser-engraved onto self-adhesive paper. (**c**) Positive paper masters formed by removing the complementary parts for PDMS molding. (**d**) Fabricated prototypes of the PDMS-based microfluidic devices.

The fabrication process of the PDMS-based microfluidic device was carried out by adopting a straightforward and cost-efficient method, which was elaborated in our previous research work [22]. A Schematic flow-focusing geometry for the positive master was designed with the aid of AutoCAD 2017 software (Autodesk, Inc., San Rafael, CA, USA) to prototype the PDMS microfluidic device, as illustrated in Figure 1a. The distance between the double flow-focusing junctions was set to 10 mm, while the width of all the microchannels was maintained at 500 µm. Self-adhesive paper with a thickness of 900 µm, sourced from Chengdu, China, was manually affixed to a glass slide. Instead of using a CNC (Computerized Numerical Control) machine, a laser machine (C180Ⅱ, GCC LaserPro, E Valley Blvd. Walnut, CA, USA) was utilized to fabricate a patterned positive master from the aforementioned self-adhesive paper, as shown in Figure 1b. After the fabrication process, the entire cross-sectional dimensions of the positive master were 900 × 350 µm (depth × width), as depicted in Figure 1c. Subsequently, a degassed mixture of PDMS and initiators in a ratio of 10:1 was poured onto the positive master and cured at 55 °C for 90 min. After the solidification process was fully completed, the PDMS slab was carefully peeled off from the master, cut into the desired shape, and holes were punched. Finally, after being treated in oxygen plasma (TS-SY05, tonsontec, Shenzhen, China), the PDMS slab was hermetically sealed onto a glass substrate to finish the microfluidic device, as shown in Figure 1d.

### 2.3. Characterization of Hydrogel Microspheres

**Figure 2:**
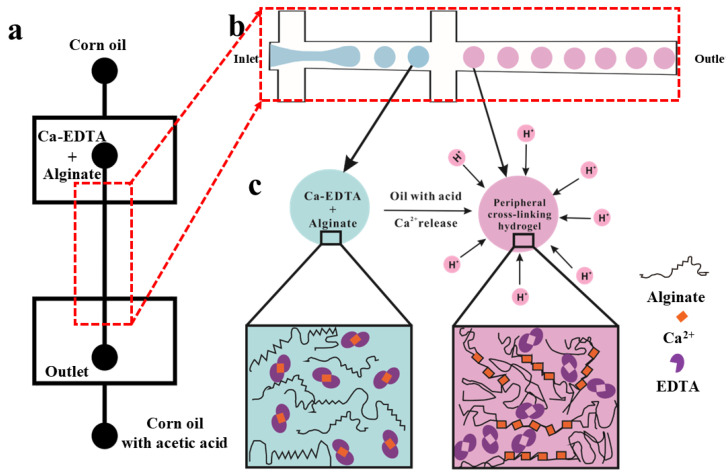
The microfluidic formation of homogeneously sized alginate microspheres encompasses several crucial components: (**a**) A symmetric flow-focusing pattern of the microfluidic device, designed using Auto CAD software. (**b**) An enlarged illustration of the local flow-focusing structure in (**a**). (**c**) A schematic diagram depicting the process of alginate cross-linking. Herein, the introduction of acid at the flow-focusing structure triggers the complete formation of cross-linked microspheres as calcium ions are released from the calcium–EDTA complex solution.

Figure 2 shows the illustration of the microfluidic homogeneously sized alginate microspheres formation in a symmetric flow-focusing device. The flow rates for corn oil, calcium–EDTA hydrogel, and acetic oil during the formation of hydrogel microspheres were set to 2 µL/min, 30 µL/min, and 30 µL/min, respectively. After synthesis, the microspheres were separated, washed with deionized water, and subsequently frozen. Distribute the sample on an in-situ temperature-controlled stage and allow it to cool to −20 °C for solidification. Transfer the solidified sample into the scanning electron microscope (SEM) sample chamber. Observe the surface morphology of the sample under an operating voltage of 15 kV in a Phenom Pharos G2 Desktop FEG-SEM (Thermo Fisher Scientific, Waltham, MA, USA), and capture images at magnifications of 180×, 430×, 1000×, and 1500×, respectively, enabling high-resolution characterization of both surface features and microstructural details.

## 3. Results and Discussion

### 3.1. Formation of Hydrogel Microsphere

**Figure 3:**
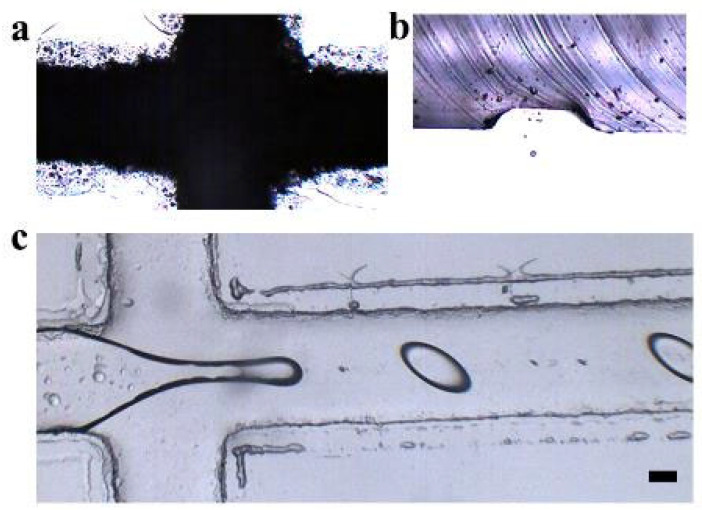
Characteristic features of the PDMS microfluidic device. (**a**) Microscopic image of paper master fabricated by laser cutting. (**b**) Cross-section of the PDMS microfluidic channel. (**c**) Droplet formation in this microfluidic device. Scale bars: 100 µm (applies to (**a**–**c**)).

In this work, the paper-based master fabrication strategy fundamentally alters cost structures by three aspects: (i) Eliminating cleanroom requirements. (ii) Reducing mold material costs by 99% compared to SU-8 lithography. (iii) Enabling rapid prototyping (≤2 h per device vs. 24+ h for PDMS devices), shown in Table 2. Table 3 shows the details of the comparison between the designed dimension and the fabricated dimension. In our proposal method, the positive masters were fabricated decreasingly by laser cutting. Figure 3a shows the microscopy image of the self-adhesive paper positive master. Figure 3b shows the cross-sectional microscopy image of the PDMS microfluidic channels, of which the geometry is trapezoid. Figure 3c shows the prototype microfluidic device for droplet formation.

**Figure 4:**
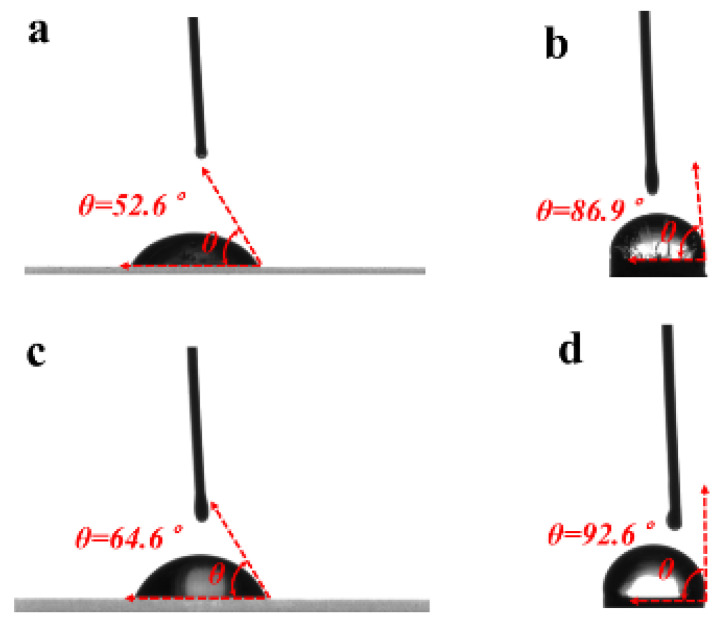
Contact angle measurements on glass and silicone substrates. (**a**) Corn oil droplet on glass. (**b**) Corn oil droplet on silicone. (**c**) Sodium alginate solution containing Ca-EDTA droplet on glass. (**d**) Sodium alginate solution containing Ca-EDTA droplet on the surface of silicone substrate.

The Ca-EDTA complex was prepared by mixing 5 M CaCl_2_ and 0.5 M EDTA at a 1:64 volume ratio, followed by pH adjustment to 7.2 using 4 M NaOH. An aqueous 2% (*w*/*v*) sodium alginate solution was then combined with the pH-adjusted Ca-EDTA complex in equal volumes, yielding a final 1% (*w*/*v*) sodium alginate solution containing Ca-EDTA. Separately, a 2% (*v*/*v*) acetic acid solution in corn oil was prepared by mixing at a 1:49 volume ratio. According to the Young–Laplace equation, interfacial tension (γ) of corn oil and sodium alginate solution containing Ca-EDTA was measured easily using the pendant drop method on glass and silicone substrates (Figure 4), respectively, and calculated by Young’s Equation (1), as shown in Table 1. Figure 4a shows the contact angle measurement of corn oil on glass, while Figure 4b shows the corresponding measurement on silicone. Similarly, Figure 4c presents the contact angle of sodium alginate solution containing Ca-EDTA on glass, with Figure 4d showing the silicone substrate measurement. The work of interfacial tension between corn oil and sodium alginate solution containing Ca-EDTA was calculated using the classical Girifalco–Good Equation (2).

(1) $$\gamma_{SV}=\gamma_{SL}+\gamma_{LV}\cos\theta$$

Among them, $\gamma_{SV}$ is the surface tension at the solid-vapor interface, $\gamma_{SL}$ represents the surface tension at the solid–liquid interface, and $\gamma_{LV}$ is the surface tension at the liquid-vapor interface, θ is the contact angel.

(2) $$\gamma_{LL}=\gamma_{CO}+\gamma_{SA}-2\phi \sqrt{\gamma_{CO}\gamma_{SA}}$$

While $\gamma_{LL}$ is the surface tension at the immiscible liquid–liquid interface, $\gamma_{CO}$ and $\gamma_{SA}$ denote the surface tension of corn oil and sodium alginate solution containing Ca-EDTA, while ϕ is coefficient of the interaction parameter. As shown in Table 1, ϕ = 0.61, where $\gamma_{LL}$ is calculated to 66.49 mN/m using Equations (1) and (2).

**Figure 5:**
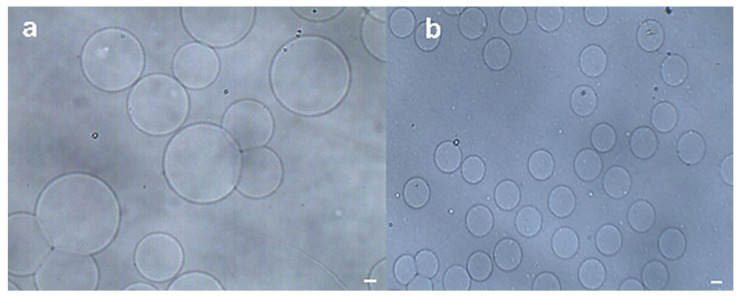
Microfluidic synthesis of surfactant-free alginate microspheres. (**a**) Bright-field microscopy image of non-uniform alginate microspheres generated in a single-channel flow focusing microfluidic device without surfactant. (**b**) Corresponding microspheres produced via symmetric dual-channel flow-focusing microfluidic emulsification, demonstrating uniform surfactant-free alginate droplet templating. Scale bars: 100 µm.

Figure 5 illustrates the microfluidic device used for the formation of alginate microspheres. A calcium–EDTA complex dissolved in sodium alginate solution served as the dispersed phase, while surfactant-free corn oil functioned as the continuous phase, generating non-uniform alginate microspheres in a single-channel flow-focusing geometry, as shown in Figure 5a. Interestingly, in this symmetric flow-focusing device, droplets formed at the primary junction while acetic acid/corn oil mixture (1:49 *v*/*v*) was introduced at the secondary junction to prevent droplet coalescence through rapid interfacial gelation, as it depicted in Figure 5b. The surfactant-free system enabled effective generation of monodisperse sodium alginate/Ca-EDTA microspheres, yielding an emulsion with narrow size distribution (CV < 5%, n = 50).

At another flow-focusing junction downstream, acetic acid (2% *w*/*w*) dissolved in corn oil was introduced and mixed with the stream of monodisperse droplets. The H^+^ ions from the acidic oil readily diffused into the aqueous Na-alginate droplets. Notably, the acidic oil was mixed with the corn oil downstream to stabilize the Na-alginate droplets against coalescence [23], thereby preserving the homogeneous size of the spherical alginate microspheres.

### 3.2. Separation of Hydrogel Microsphere

Figure 5a visually depicts the hydrogel microspheres coalescence without any surfactant in continuous oil phase, which were generated using single flow-focusing microfluidic device. To isolate the hydrogel microspheres from the corn oil, the emulsions were transferred into a Petri dish containing an aqueous solution. The separation of the hydrogel microspheres from the oil phase was initially achieved by placing the Petri dish on a micro-plate shaker (QB-8001, Mini shaker, Haimen, China) and subjecting it to an oscillating motion at 300 rpm/min. Notably, these microspheres demonstrated a remarkable ability to migrate rapidly from the oil phase to the aqueous phase in this symmetry flow-focusing microfluidic system. In this step, the droplet coalescence can be prevented efficiently for the mass production of monodisperse microspheres, as evidenced in Figure 5a,b. Once the microspheres settled at the bottom of the aqueous solution, excess oil floating on the aqueous phase was easily removed via aspiration without requiring demulsifiers. The results demonstrate that this two-stage washing reduced residual oil content to <7% *w*/*w* (n = 6)—closely approaching the <2.1% *w*/*w* achieved by traditional demulsification methods. This validates the superiority of our approach in eliminating complex demulsification steps while ensuring minimal oil residue under mild conditions. Remarkably, the entire extraction process was completed in just 20 min, with minimal cost, highlighting its efficiency and economic viability, as summarized in Table 4.

**Figure 6:**
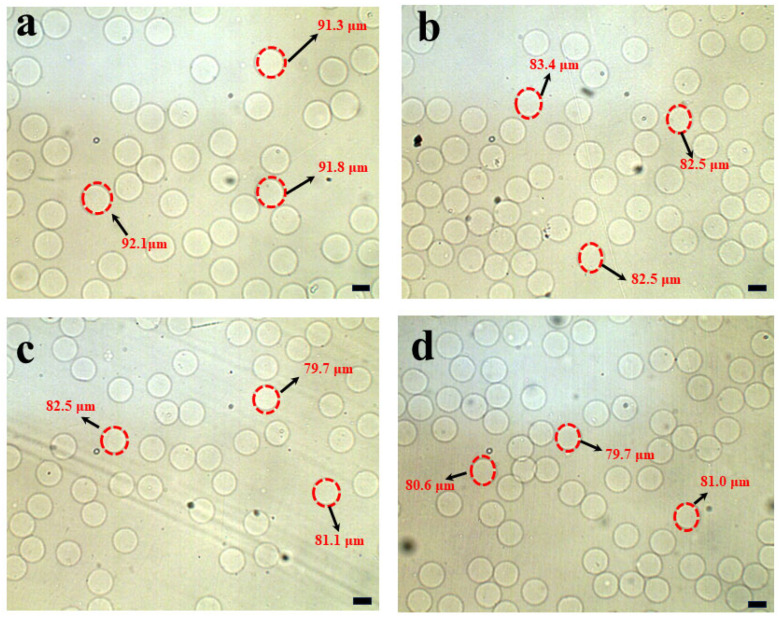
Phase-contrast microscopic images depicting the alginate microspheres’ radius after the demulsification process are presented. In these experimental configurations, the flow rates of the disperse alginate–calcium–EDTA solution were firmly set at 2 µL/min. (**a**) With regard to the specific microfluidic device, in order to regulate the droplet size, the flow rate of the continuous oil phase was adjusted to 30 µL/min. (**b**) The flow rate of the continuous oil phase was established at 35 µL/min. (**c**) The continuous oil phase flowed at a rate of 45 µL/min. (**d**) The flow rate of the continuous oil phase was maintained at 50 µL/min. Scale bars: 100 µm.

**Figure 7:**
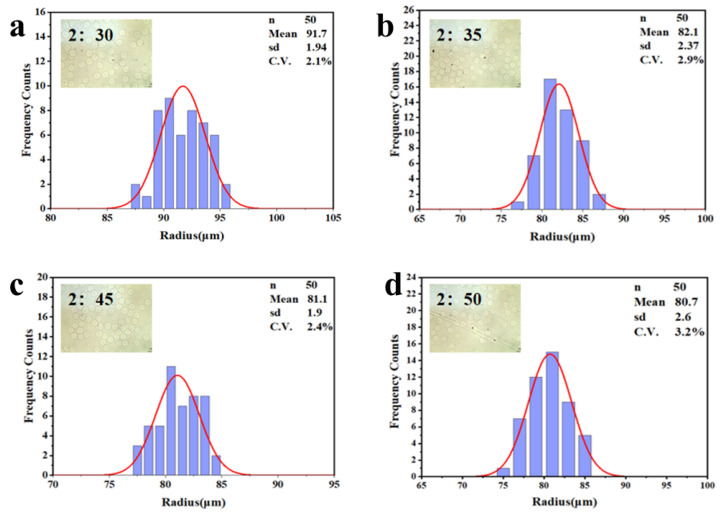
Size distribution of the alginate hydrogel microspheres shown in (**a**–**d**) with the constant flow rate of disperse alginate phase 2 µL/min, and the varied flow rates of the continuous oil phase from 30 µL/min to 50 µL/min, respectively.

In the preparation step, the calcium–EDTA complex was meticulously prepared by combining equal volumes of an aqueous calcium chloride solution and a disodium–EDTA solution. The pH of the resulting mixture was carefully adjusted to 7.2 to ensure the high stability of the complex. As illustrated in Figure 6 and Figure 7, the size of the hydrogel microspheres could be precisely controlled by modulating the flow rate of the continuous phase, specifically corn oil. Under the experimental conditions where the calcium–EDTA complex concentration in the alginate medium was fixed at 1 wt% and the corn oil contained 2% *w*/*w* acetic acid, spherical hydrogel microspheres with radius ranging from 91.7 ± 1.94 µm to 80.7 ± 2.60 µm were successfully fabricated by varying the continuous phase flow rate, as shown in Figure 7a–d. During the experiments, the flow rate of the alginate solution was held constant at 2 µL/min, while the flow rate of the corn oil was systematically increased from 30 µL/min, 35 µL/min, 45 µL/min, and 50 µL/min, respectively. Notably, the flow rate of the acidic oil phase was accompanied with the same flow rate of corn oil at 30 µL/min, 35 µL/min, 45 µL/min, and 50 µL/min, respectively. As clearly demonstrated in Figure 6a–d, this adjustment resulted in a significant reduction in emulsion diameter, highlighting a direct correlation between the corn oil flow rate and the size of the hydrogel microspheres. The synthesized microspheres demonstrated remarkably low size polydispersity, as evidenced by an exceptional coefficient of variation (C.V.) below 5%. This surfactant- and demulsifier-free fabrication strategy achieves significant cost efficiencies through two synergistic mechanisms: (1) Elimination of conventional post-synthesis purification processes (typically requiring 6–8 washing cycles for surfactant and oil residue removal), and (2) Direct omission of interfacial stabilizers, which conventionally account for 99% of total production costs in traditional methodologies.

### 3.3. Surface Morphologies and Microstructures of Hydrogel Microsphere

**Figure 8:**
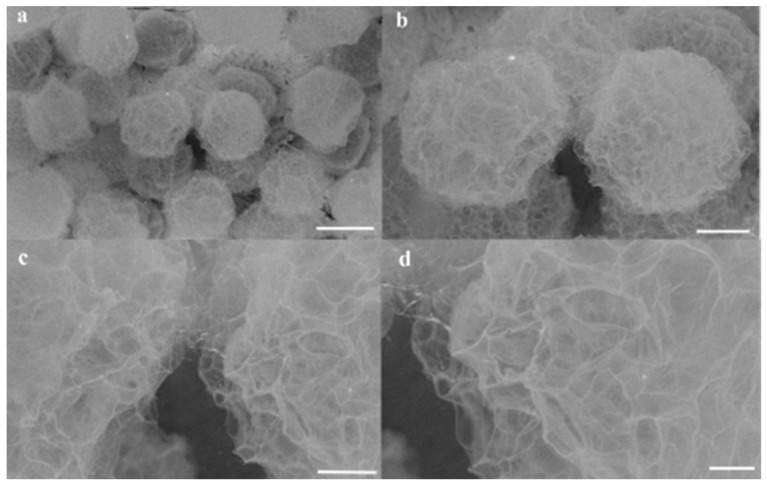
SEM characterization of alginate hydrogel microspheres synthesized under fixed dispersed-phase flow conditions. (**a**–**d**) SEM images of microspheres generated with a dispersed alginate phase flow rate of 2 µL/min and a continuous oil phase flow rate of 30 µL/min. Magnification and scale bars: (**a**) 180× (scale bar: 100 µm), (**b**) 430× (scale bar: 40 µm), (**c**) 1000× (scale bar: 20 µm), (**d**) 1500× (scale bar: 10 µm).

Figure 8 shows representative SEM images of the microspheres at magnifications ranging from 180× to 1500×. The surface morphology reveals distinct roughness and uniformity across the microspheres. At higher magnifications (Figure 8a–d), high-resolution imaging highlights numerous porous regions on the surfaces. This porous architecture facilitates rapid permeation of the solution and H^+^ ions into the microspheres, enabling complete solidification within 15 min.

## 4. Conclusions

In summary, we present an innovative two-step microfluidic approach for fabricating monodisperse alginate hydrogel microspheres with exceptional spherical uniformity. By employing the calcium–EDTA complex as a crosslinking precursor and utilizing pH-triggered Ca^2+^ ion release, we achieve consistent and homogeneous gelation of the microspheres. This method offers the distinct advantage of enabling precise control over the physical properties of the microgels. Through careful adjustment of the crosslinker concentration and alginate chain characteristics, the properties of the microspheres can be finely tailored to meet specific requirements. Furthermore, our results demonstrate that this process is not only cost-effective but also highly adaptable for use in resource-limited settings.

## Figures and Tables

**Table 1:** Densities, viscosities and the interfacial tension of the fluids used in this work, measured at 25 °C.

Fluid	Density (ρ (g/mL^−1^))	Dyn. Viscosity (η (mPa·s))	Interfacial Tension (mN m^−1^)
Corn oil	0.96	1.4	85.5
Acetic corn oil (2%)	0.95	1.8	-
Alginate solution (1%)	1.68	4.4	72.0

**Table 2:** SU-8 vs. paper masters for PDMS molding: a feature comparison.

Master Type	Time (in Hour)	Price ($)	Minimum Width (in mm)
SU-8	>24	~700	≥0.001
Paper	~2	~0.03	≥0.080

**Table 3:** The average and standard deviation (n = 3) of microchannel width for each step.

Master Type	Width of Master (in µm)	PDMS Prototyping (in µm)
Designed size	500	399.5 ± 4.7
Fabricated size	399.5 ± 4.7	339.8 ± 9.7

**Table 4:** The distinct comparative advantages of the two-stage approach when contrasted with the represent methods.

Master	Surfactant	Demulsification	Ref.
Glass tube	F-127	Isopropyl alcohol	[1]
SU-8	Span 80	PFO	[2,16]
SU-8	Lecithin	Hexane/Hexadecane	[6]
SU-8	PFPE-PEG	PFO	[8,11,14]
SU-8	Picosurf^TM^	1H,1H-Perfluorooctan-1-ol	[12]
SU-8	neat 008-uorosurfactant	PFO	[13]
SU-8	Krytox–PEG–Krytox	PFO	[15]
Paper	Without addition	Without addition	This work

SU-8: SU-8 2050 photoresist. F-127: pluronic F-127. PFPE-PEG: perfluorinated polyetherspolyethyleneglycol. PFO: 1H,1H,2H,2H-Perfluoro-1-octanol.

## Data Availability

The original contributions presented in the study are included in the article, further inquiries can be directed to the corresponding authors.
